# Persistent hypercalcaemia associated with two pathogenic variants in the *CYP24A1* gene and a parathyroid adenoma—a case report and review

**DOI:** 10.3389/fendo.2024.1355916

**Published:** 2024-04-11

**Authors:** Dorota Leszczyńska, Alicja Szatko, Julia Latocha, Magdalena Kochman, Maria Duchnowska, Anna Wójcicka, Waldemar Misiorowski, Wojciech Zgliczyníski, Piotr Glinicki

**Affiliations:** ^1^ Department of Endocrinology, Centre of Postgraduate Medical Education, Warsaw, Poland; ^2^ EndoLab Laboratory, Centre of Postgraduate Medical Education, Warsaw, Poland; ^3^ Students’ Scientific Group Affiliated with the Department of Endocrinology, Centre of Postgraduate Medical Education, Warsaw, Poland; ^4^ Warsaw Genomics, Warsaw, Poland; ^5^ Fundacja Wiedzieć Więcej, Warsaw, Poland

**Keywords:** hypercalcaemia, *CYP24A1* gene, vitamin D, primary hyperparathyroidism, 25(OH)D_3_, 1,25(OH)_2_D_3_, 24,25(OH)_2_D_3_, 24-hydroxylase deficiency

## Abstract

**Introduction:**

24-Hydroxylase, encoded by the *CYP24A1* gene, is a crucial enzyme involved in the catabolism of vitamin D. Loss-of-function mutations in *CYP24A1* result in PTH-independent hypercalcaemia with high levels of 1,25(OH)_2_D_3_. The variety of clinical manifestations depends on age, and underlying genetic predisposition mutations can lead to fatal infantile hypercalcaemia among neonates, whereas adult symptoms are usually mild.

**Aim of the study:**

We report a rare case of an adult with primary hyperparathyroidism and loss-of-function mutations in the *CYP24A1* gene and a review of similar cases.

**Case presentation:**

We report the case of a 58-year-old woman diagnosed initially with primary hyperparathyroidism. Preoperatively, the suspected mass adjoining the upper pole of the left lobe of the thyroid gland was found via ultrasonography and confirmed by 99mTc scintigraphy and biopsy as the parathyroid gland. The patient underwent parathyroidectomy (a histopathology report revealed parathyroid adenoma), which led to normocalcaemia. After 10 months, vitamin D supplementation was introduced due to deficiency, and the calcium level remained within the reference range. Two years later, biochemical tests showed recurrence of hypercalcaemia with suppressed parathyroid hormone levels and elevated 1,25(OH)_2_D_3_ concentrations. Further investigation excluded the most common causes of PTH-independent hypercalcaemia, such as granulomatous disease, malignancy, and vitamin D intoxication. Subsequently, vitamin D metabolites were measured using LC–MS/MS, which revealed high levels of 25(OH)D_3_, low levels of 24,25(OH)_2_D_3_ and elevated 25(OH)_2_D_3_/24,25(OH)_2_D_3_ ratios, suggesting a defect in vitamin D catabolism. Molecular analysis of the *CYP24A1* gene using the NGS technique revealed two pathogenic variants: p.(Arg396Trp) and p.(Glu143del) (rs114368325 and rs777676129, respectively).

**Conclusions:**

The diagnostic process for hypercalcaemia becomes complicated when multiple causes of hypercalcaemia coexist. The measurement of vitamin D metabolites using LC–MS/MS may help to identify carriers of *CYP24A1* mutations. Subsequent molecular testing may contribute to establishing the exact frequency of pathogenic variants of the *CYP24A1* gene and introducing personalized treatment.

## Introduction

The active form of vitamin D (1,25(OH)_2_D_3_ or calcitriol), in addition to parathyroid hormone (PTH) and fibroblast growth factor 23 (FGF23), is critical for maintaining calcium haemostasis (1). Primary hyperparathyroidism (PHPT) and malignancy are the most common causes of hypercalcaemia. Among the less frequent aetiologies, vitamin D-mediated hypercalcaemia should be considered.

**Figure 1:**
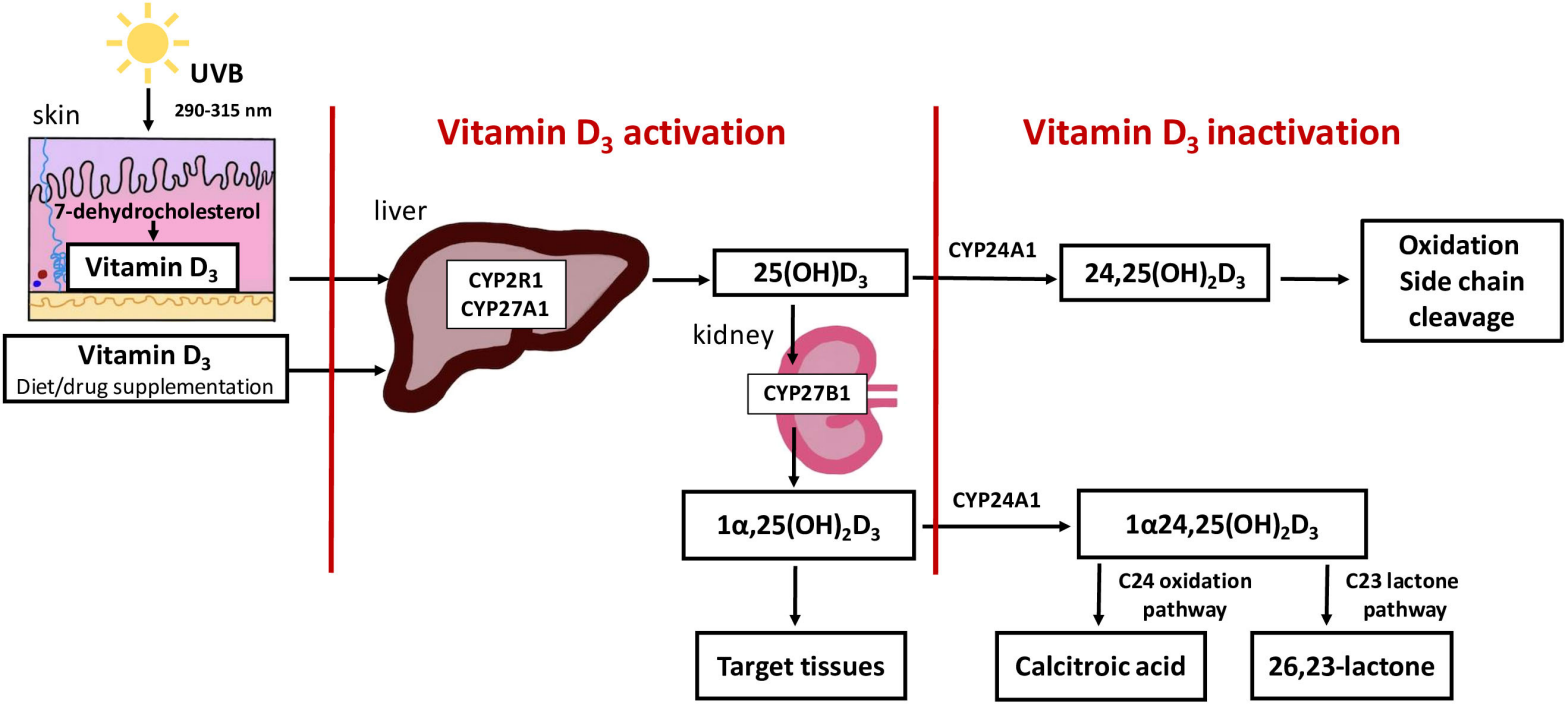
Overview of vitamin D synthesis, activation and inactivation. The main source of vitamin D is skin synthesis from 7-dehydrocholestrol (7-DHC), however, it can also be obtained from the diet. For full hormonal activity, vitamin D_3_, requires two-stage hydroxylation. First, 25 hydroxylase generates 25(OH)D_3_ in the liver. The second hydroxylation by 1-alpha-hydroxylase (CYP2781, Cytochrome P450 Family 27 Subfamily B Member 1) leads to the conversion of 25(OH)D_3_ to the biologically active metabolite of vitamin D (1,25(OH)_2_D_3_). This process occurs mainly in the kidneys. 1,25(OH)_2_ D_3_ and 25(OH)D_3_ are inactivated by 24-hydroxylase (CYP24A1, cytochrome P450 family 24 subfamily a member 1) the key enzymes involved in vitamin D catabolism. The direct products of the CYP24A1 reaction are 24,25(OH)_2_D_3_ and 1,24,25(OH)_2_D_3_, which are further converted to calcitroic acid or a 26,23- lactone derivative.

The main source of vitamin D is skin synthesis from 7-dehydrocholestrol (7-DHC); however, it can also be obtained from the diet. For full hormonal activity, vitamin D_3_ requires two-stage hydroxylation. First, 25 hydroxylase generates 25(OH)D_3_ in the liver. The second hydroxylation by 1-alpha-hydroxylase (CYP27B1, Cytochrome P450 Family 27 Subfamily B Member 1) leads to the conversion of 25(OH)D_3_ to the biologically active metabolite of vitamin D (1,25(OH)_2_D_3_) (2). This process occurs mainly in the kidneys, but *CYP27B1* is also expressed at several external sites (3). 1,25(OH)_2_D_3_ and 25(OH)D_3_ are inactivated by 24-hydroxylase (24HD) (cytochrome P450 family 24 subfamily a member 1 (CYP24A1)), the key enzymes involved in vitamin D catabolism. The direct products of the CYP24A1 reaction are 24,25(OH)_2_D_3_ and 1,24,25(OH)_2_D_3_, which are further converted to calcitroic acid destined for biliary excretion (4) (**Figure 1**). The activity of CYP24A1 is regulated by numerous biochemical factors. Hypocalcaemia and PTH downregulate *CYP24A1* expression and enzymatic activity (5). The opposite stimulating effect is caused by 1,25(OH)_2_D_3_ and FGF23, together with its coreceptor α-Klotho protein (5).

The concentration of 25(OH)D, the best indicator of vitamin D status, can be measured by numerous diagnostic methods. The following immunoassays were used: chemiluminescence immunoassay (CLIA), electrochemiluminescence immunoassay (ECLIA), vitamin D binding protein (DBP-based assays), high-performance liquid chromatography (HPLC) and liquid chromatography with tandem mass spectrometry (LC−MS/MS). However, the ‘gold standard’ and reference technique that allows for the most reliable assessment of many vitamin D metabolites simultaneously is LC–MS/MS (6).

Excessive vitamin D or calcitriol ingestion, ectopic production of 1,25(OH)_2_D_3_ in granulomatous or lymphoproliferative disease and genetically determined dysregulation of vitamin D metabolism may lead to PTH-independent hypercalcaemia (7). There are two known underlying genetic mechanisms resulting from either mutations in *CYP24A1*encoding 24HD leading to an inability to deactivate 25(OH)D_3_ and 1,25(OH)_2_D_3_ or in the sodium phosphate cotransporter IIa gene (SLC34A1, Solute Carrier Family 27 Subfamily B Member 1), resulting in excessive 1,25(OH)_2_D_3_ biosynthesis secondary to chronic phosphate wasting (8). Both genetic disorders cause idiopathic infantile hypercalcaemia (HCINF), which is associated with hypersensitivity to vitamin D, especially in patients receiving vitamin D supplementation. In patients with *CYP24A1* mutations, laboratory tests revealed an increased 25(OH)D_3_/24,25(OH)_2_D_3_ ratio. This investigation has been recommended as a screening tool whenever genetic causes of hypercalcaemia are considered (9).

## Aim of the study

The aim of this study was to determine the importance of determining vitamin D metabolites in the differential diagnosis of hypercalcaemia and the possibility of co-occurring various causes of hypercalcaemia. In this study, we present a rare case of an adult patient with PHPT and hypercalcaemia caused by two heterozygous pathogenic *CYP24A1* variants (confirmed by next-generation sequencing [NGS]) with a review of reported cases of patients with hypercalcaemia associated with pathogenic variants of *CYP24A1* and concomitant PHPT.

## Case presentation

### Clinical data

A 58-year-old woman was referred to the Endocrinology Unit in 2019 due to incidental findings of hypercalcaemia. Her medical history included hypertension, stage 3a chronic kidney disease (CKD), and carpal tunnel syndrome treated surgically. She had no family history of endocrine diseases or nephrolithiasis.

### Biochemical tests

**Figure 2:**
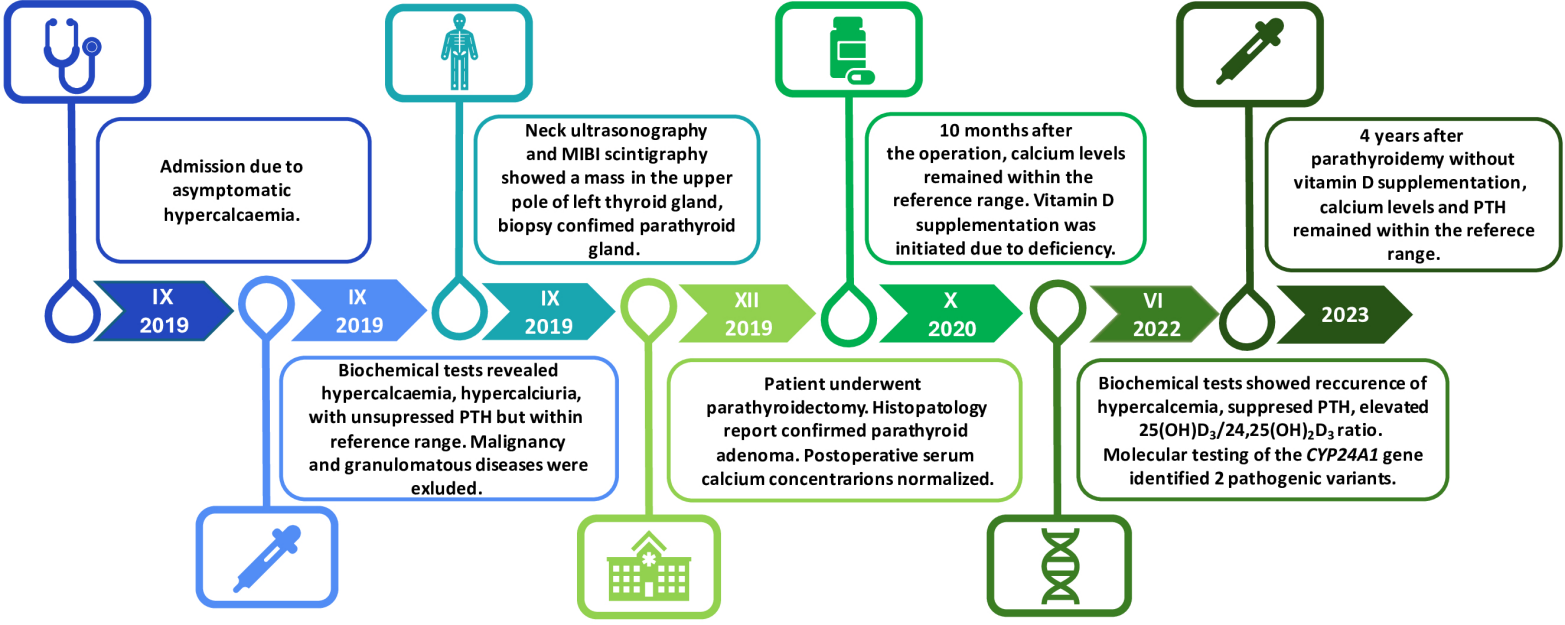
Timeline summarization of the patient’s clinical course. Asymptomatic hypercalcaemia was incidentally found in 2019. The same year, primary hyperparathyroidism (PHPT) was diagnosed, and the presence of suspected lesion was confirmed in the imaging studies. The patient underwent parathyroidectomy which led to normocaicaemia. After 10 months, vitamin D supplementation was initiated due to deficiency. In 2022, biochemical tests revealed recurrence of hypercalcaemia with suppressed PTH concentration. After the exclusion of the common causes of PTH-independent hypercalceamia, the presence of two heterozygous loss-of-function mutations in the *CYP24A1* gene was confirmed in Next-Generation Sequencing (NGS). After discontinuation of vitamin D supplementation, calcium and PTH concentrations normalized. PTH-parathormone; MIBI -technetium-99m-sestamibi scintigraphy; *CYP24A1*- cytochrome P450 family 24 subfamily a member 1.

A laboratory test revealed hypercalcaemia, hypercalciuria, and unsuppressed PTH levels (27 pg/mL, reference range, 15-65 pg/mL) (**Table 1**; **Figure 2**).

**Table 1:** Laboratory results presented in chronological order.

Analysed parameter, units	2018^a^	2020^b^	2022^c^	2023^d^	Reference range
Corrected calcium, mmol/L	3.0	2.48	2.88	2.51	2.2-2.55
Phosphate, mg/dL	0.81	1.1	0.93	1.06	0.81-1.45
25(OH)D_3_, ng/mL	34.8	16.4	34.2	27.4	30-50
1,25(OH)_2_D_3_, pg/mL	72.0	48.0	90.6	64.4	19.9 – 79.3
Parathormone, pg/mL	27.0	19.0	7.7	15.0	15-65
Creatinine, mg/dL GFR, ml/min/1,73 m^2^	1.05 54.6	1.08 52.5	1.38 39.3	1.26 43.4	0.5-0.9 >90
Calcium in 24h urine collection, mmol/24hr	11.97	5.13	7.81	N/A	2.5-7.5

a. baseline results before parathyroidectomy; b. 10 months after parathyroidectomy without vitamin D supplementation; c. 3 years after parathyroidectomy with vitamin D 4000 IU daily; d. 4 years after parathyroidectomy without vitamin D supplementation; GFR- glomerular filtration rate; N/A - not available.

### Imaging

Computed tomography (CT) scans of the chest, abdomen and pelvis were conducted and showed no signs of malignancy or granulomatous disease.

Neck ultrasonography and technetium 99m sestamibi (MIBI) scintigraphy revealed a mass in the upper pole of the left thyroid gland lobe, which was confirmed to be the parathyroid gland after biopsy via immunohistochemical staining (**Figure 2**).

There were no abnormalities in the densitometry results (T score of the lumbar spine 1.1; T score of the proximal femur -0.2; T score of the radius 0.5). However, an X-ray revealed advanced subperiosteal bone resorption in the fingers and bone loss in the thoracic spine and clavicles. There were no renal stones or fractures.

### Treatment and outcomes

The patient underwent effective parathyroidectomy, after which the calcium level was normalized (2.45 mmol/L, reference range, 2.2-2.55 mmol/L). Histopathology confirmed parathyroid adenoma.

Ten months after the operation, postoperative assessment revealed a normal calcium concentration (2.48 mmol/L), but vitamin D deficiency (16.4 ng/mL, reference range, 30-50 ng/mL); thus, supplementation with cholecalciferol was administered (4000 IU/daily) (**Table 1**; **Figure 2**). The patient did not attend her follow-up appointments for two years. In 2022, a laboratory test showed hypercalcaemia and hypercalciuria recurrence, which was initially identified as recurrent primary hyperparathyroidism. However, blood tests revealed PTH suppression with elevated 1,25(OH)_2_D_3_ concentrations (**Table 1**; **Figure 2**).

The patient was screened for granulomatous disease and hypercalcaemia of malignancy again, and the results were negative.

Vitamin D metabolites were measured using LC–MS/MS, which revealed high 25(OH)D_3_ (72.62 ng/mL) and low 24,25(OH)_2_D_3_ (0.09 ng/mL) concentrations and an elevated 25(OH)D_3_/24,25(OH)_2_D_3_ ratio 806,9 (reference range, 7.0-23.6), suggesting a defect in vitamin D catabolism.

### Molecular testing

The genetic testing of the *CYP24A1* gene was conducted using the NGS technique, and two pathogenic variants were identified NM_000782.5:c; 1186C>T(p.Arg396Trp, rs114368325) and NM_000782.5:c; 428_430del (p.Glu143del, rs777676129). Both variants are classified as pathogenic/likely pathogenic, and associated with hypercalcemia in the available databases, including ClinVar NIH (ClinVar archives, National Institutes of Health). Due to the unavailability of family members, it was impossible to assess whether the variants were located in the same of different alleles of *CYP24A1*, what would be a direct proof of their dominant or recessive involvement in development of the observed symptoms.

The recommendation was to discontinue vitamin D supplementation, maintain adequate hydration and avoid excessive sunlight exposure. Follow-up evaluation showed normalization of calcium and PTH concentrations (**Table 1**).

## Discussion

Hypercalcaemia is a common clinical abnormality. PTH measurement is the key initial test for differentiating between PTH-dependent and PTH-independent hypercalcaemia.

A high or inappropriately normal PTH level in relation to a high calcium concentration is typical for PTH-dependent causes. Among them, the most common is PHPT, whose reported incidence varies between 0.2% and 1.3% across the population (10). In PTH-independent hypercalcaemia, the PTH level is appropriately suppressed for hypercalcaemia status. Malignancy is the most common disorder in this population. Among other causes of PTH-independent hypercalcaemia, vitamin D-mediated hypercalcaemia should be considered.

Currently, in many countries where the evaluation of serum calcium levels has become routine, PHPT appears to be the most common cause of hypercalcaemia and is identified most frequently in the initial asymptomatic stage (11). A growing prevalence of PHPT results in the identification of unusual cases of overlapping PTH-dependent and PTH-independent causes of hypercalcaemia. We described a woman with asymptomatic mild hypercalcaemia and ambiguous low-normal PTH, which made the initial classification of hypercalcaemia difficult. For this reason, in the differentiation, we considered both PTH-dependent and PTH-independent causes of hypercalcaemia. After excluding malignancy and granulomatous diseases, PHTP was diagnosed based on imaging studies and parathyroid biopsy. The patient underwent successful parathyroidectomy. Only the recurrence of PTH-independent hypercalcaemia after vitamin D supplementation revealed the other cause of hypercalcaemia, which turned out to be a genetic disorder of vitamin D catabolism.

Pathogenic mutations in the *CYP24A1* gene clinically manifest as infantile hypercalcaemia-1 (HCINF1, OMIM 143880), a rare disorder linked with a disturbance in vitamin D degradation (12). HCINF1 is classically characterized by severe symptoms such as vomiting, polysomnia, hypotonia, constipation, failure to thrive, and renal stone disease (13). The first case reports of HCINF1 were published in the 1950s, when approximately 200 cases of PTH-independent hypercalcaemia in infants were noted in the United Kingdom because of the wide use of formula milk enriched with increased doses of vitamin D (up to 4000 International Units (IU)) (14, 15). Although the majority of affected children developed mild hypercalcaemia not associated with other syndromic features (HCINF1), the percentage of fatal cases was remarkable, and some affected children had multisystem disorders, which were later described as Willams–Beuren syndrome (OMIM 194050) (12, 16). At that time, vitamin D dietary intake was identified as a precipitating factor of HCINF1, yet the exact pathogenesis has remained unknown (12, 14).

Only recently, the structure of the *CYP24A1* gene and the pathways involved in vitamin D metabolism leading to HCINF1 were revealed. In 1991, Ohyama et al. described the isolation of complementary deoxyribonucleic acid (cDNA) from a rat kidney cDNA library utilizing antibodies specific for the enzyme (17). Two years later, Chen et al. published a paper on the isolation, sequencing and expression of cDNA encoding the human 24HD (18). In the same year, Hahn et al. independently isolated human kidney cDNA encoding vitamin D 24HD (19). In 2011, Schlingmann et al. confirmed that HCINF1 is mutated in *CYP24A1* based on 8 cases of infants receiving vitamin D supplementation (either daily or bolus doses) (12). The affected children were homozygotes or compound heterozygotes for nonsense or missense mutations of *CYP24A1* (inherited as autosomal recessive trait), leading to complete loss of function of 24HD confirmed in the transfected eukaryotic cell line (12). Since then, multiple pathogenic variants (PVs) in the *CYP24A1* gene have been identified, revealing that the spectrum of biallelic variants (either homozygous or compound heterozygous) ranges from severe hypercalcaemia in infants to mild hypercalcaemia in adults (20–23). Heterozygotes usually present with milder or asymptomatic phenotypes (24). However, in the case of a 44-year-old patient with intermittent hypercalcaemia and two intron−exon splice junction mutations (IVS5 + 1G>A and IVS6–2A>G) of *CYP24A1*, an analysis of family members suggested autosomal dominant inheritance with partial penetrance (25). Furthermore, the clinical phenotype is dependent not only on the PV but also on vitamin D intake, sunlight exposure or pregnancy (12, 26, 27). It is known that 1,25(OH)_2_D_3_ is elevated during normal pregnancy, which additionally promotes hypercalcaemia in women with disturbed calcitriol catabolism associated with *CYP24A1* mutation (5, 28).

Hypercalcaemia related to the presence of PVs in the *CYP24A1* gene is often overlooked. Although epidemiological data concerning the prevalence of PVs of the *CYP24A1* gene are scarce, the estimated frequency of deleterious minor alleles is 0.140 (29). Thus, the calculated frequency (using the Hardy−Weinberg equilibrium) of recessive disorders is 1960 per 100.000 people (29). By adding patients harbouring monoallelic mutations who may present with a clinical phenotype, the number of affected individuals is significant, and this should be considered more often in the differential diagnosis of hypercalcaemia (30, 31).

In 2016, an autosomal recessive loss-of-function mutation in the *SLC34A1* gene was identified as the second cause of infantile hypercalcaemia-2 (HCINF2, OMIM 616963) (32). Like in patients with *CYP24A1* mutations, hypercalcaemia, suppressed PTH and inappropriately high 1,25(OH)_2_D_3_ are observed. The distinguishing feature is hypophosphatemia due to renal phosphate wasting. (32)

Low PTH and high 1,25(OH)_2_D_3_ levels are specific to the whole group of endogenous causes of vitamin D related to hypercalcaemia (including HCINF, granulomatous disease and some lymphomas); therefore, 24,25(OH)_2_D_3_ assessment is crucial for differentiation. The distinguishing feature of HCINF1 is the very low concentration of 24,25(OH)_2_D_3_. Therefore, the measurement of this metabolite is the first screening tool for HCINF1, allowing to select subjects for further genetic tests. However, in healthy individuals, there is a positive linear correlation between the concentration of 25(OH)D_3_ and 24,25(OH)_2_D_3_, which results in physiological inhibition of the production of 24,25(OH)_2_D_3_ when the serum 25OHD_3_ concentration falls into the vitamin D deficiency range (33). For this reason, a more accurate parameter for expressing the absence of 24,25(OH)_2_D_3_ in HCINF1 patients is the ratio of 25(OH)D_3_ to 24,25(OH)_2_D_3_, especially because some of these patients have a low vitamin D status (34). The technique allowing a reliable assessment of 24,25(OH)_2_D_3_ is LC−MS/MS. Chromatographic separation is the reference method that allows for the precise resolution of 24,25(OH)_2_D_3_ from other vitamin D metabolites. Using the LC−MS/MS method involving derivatization with DMEQ-TAD {4-[2-(6,7-dimethoxy-4-methyl-3,4-dihydroquinoxalinyl)ethyl]-1,2,4-triazoline-3,5-dione}, a ratio greater than 80 (normal ratio, 5 to 25) allows for the identification of HCINF1 due to the *CYP24A1* mutation (34). Using extended chromatography, which resolves 24,25(OH)_2_D_3_, 25,26-(OH)_2_D_3_ and 1,25(OH)_2_D_3_, a ratio > 140 was used as the *cut-off* value (35).

To date, 6 cases of primary hyperparathyroidism coexisting with a *CYP24A1* mutation have been reported in 5 publications (**Table 2**) (36–40). The diagnosis of PHPT was made due to elevated serum calcium and inadequate nonsuppressed PTH. In most of these patients, parathyroidectomy was initially performed following the diagnosis of primary hyperparathyroidism, and after surgery, due to the persistence of hypercalcaemia, a diagnosis of 24-hydroxylase deficiency was established. Pathological examination revealed hyperplasia in the parathyroid glands in 2 patients after total or partial parathyroidectomy (37, 38). For the other subjects, adenomas were found; in one patient, a complete histopathology report was unavailable (36, 37, 39, 40). In all patients, removal of autonomous parathyroid tissue resulted in a significant decrease in the serum calcium concentration. PTH reduction may have additional value for individuals with a *CYP24A1* mutation because it eliminates the continuous stimulation of 1,25(OH)_2_D_3_ synthesis, whose catabolism is disrupted. In the present case, the relatively low concentration of PTH (27 pg/mL) before parathyroidectomy was noteworthy. Primary hyperparathyroidism is characterized by elevated calcium concentrations and a lack of portal feedback between calcium concentration and PTH secretion. Usually, the PTH level is significantly elevated but can also be within the normal range. In both situations, PTH levels are clearly inadequate for elevating serum calcium levels. However, well-documented PHPT has been reported with PTH levels as low as 20 pg/mL to 25 pg/mL, given a reference range of 10 pg/mL to 65 pg/mL (11).

**Table 2:** The summary of reported cases of concomitant primary hyperparathyroidism and mutations in the *CYP24A1* (human cytochrome P450 24 subfamily A member 1) gene leading to impaired function of 24-hydroxylas.

Authors	Year	Country	Sex	Age of diagnosis	Type of mutation	Biallelic mutation	Protein change	Serum calcium, mmol/L	PTH, pg/ml
Helmuth et al. (36)	2014	Switzerland	M	31	missense	Yes, HH	p. Arg396Trp	3.50	49
Loyer et al. (37)	2016	France	F	39	missense/ missense	Yes, CH	p.Cys380Arg /p.Leu409Ser	3.47	92
			M	51	frameshift/ missense	Yes, CH	p.Leu335Profs11 /.p.Arg396Gln	2.62 to 2.87	9, 22 then 27
David et al. (38)	2020	Belgium	M	44	missense	Yes, HH	p.Arg396Trp	2.90	36.1
Collins et al. (39)	2023	Australia	M	53	missense	Yes, HH	p.Arg396Trp	3.08	23.6
Liu et al. (40)	2023	USA	F	23	nonsense	No, Hh	p.Pro392Argfs*9	3.45	62

PTH, parathormone; M, male; F, female; HH, homozygous; CH, compound heterozygous; Hh, heterozygous.

Vitamin D and PTH are conjugated: 1,25(OH)_2_D_3_ reduces PTH directly by inhibiting its transcription and indirectly by increasing calcium absorption from the gastrointestinal tract (41). Furthermore, calcitriol induces the expression of FGF23 in bones, and FGF23 decreases PTH secretion and PTH mRNA levels (42). (42). On the other hand, PTH is the main stimulator of 1,25(OH)_2_D_3_ synthesis in the kidneys (43).

As shown in a systematic review and meta-analysis by Song et al., vitamin D supplementation in patients with PHPT and vitamin D deficiency significantly reduces PTH without causing hypercalcaemia or hypercalciuria (44). The coexistence of 24-hydroxylase insufficiency and the consequent extraordinarily high 1,25(OH)_2_D_3_ level may intensify this process. The observed concentrations of PTH in earlier described patients with HPT coexisting with *CYP24A1* mutations differed greatly from each other. Loyer et al. described 2 patients with inappropriate/high PTH levels (22 pg/mL to 92 pg/mL) (37). In the case described by Helmuth et al., the preoperative PTH concentration was 58 pg/mL (36). Another patient had PTH ranging between 36.6 pg/mL and 80.3 pg/mL during the year preceding parathyroid surgery (38). In a recent case, low-normal PTH concentrations (23.6 pg/mL) before and after the first partial parathyroidectomy were observed, and after the second parathyroid surgery, PTH became undetectable (39). The only known patient with a heterozygous pathogenic variant in *CYP24A1* had a preoperative PTH level of 63 pg/mL (40).

It is unclear whether the co-occurrence of PHPT with a *CYP24A1* mutation in these patients is a unique coincidence or is a new phenotype of *CYP24A1* mutation combined with hyperparathyroidism. The biochemical profile of patients with 24-hydroxylase deficiency-hypercalcaemia and especially high 1,25(OH)_2_D_3_ decreases not only PTH secretion but also parathyroid cell proliferation. Calcitriol reduces parathyroid volume through the suppression of the cell cycle regulator c-Myc, the suppression of transforming growth factor-α (TGF-α), and the induction of p21 (p21 protein), which is an inhibitor of the cell cycle (45). The opposite condition in which parathyroid hyperplasia occurs as a result of hyperphosphatemia, calcitriol deficiency, or hypocalcaemia is renal failure. In our patient, as well as in the other 5 described patients with the *CYP24A1* mutation and PHPT, mild renal failure was noted; however, the phosphate concentration remained low (36–39).

In most of the discussed cases, the 25(OH)D_3_:24.25(OH)_2_D_3_ ratio was determined before genetic testing (37, 38, 40). Except for one patient with a heterozygous pathogenic variant in *CYP24A1* (25(OH)D_3_:24.25(OH)_2_D_3_ ratio of 25.18), the ratios were above 100 (37, 38, 40).

In the first reported case, a homozygous loss-of-function mutation (p.Arg396Trp) in the gene encoding vitamin D 24-hydroxylase was identified; second, the most prevalent mutation in the *CYP24A1* gene was found in patients with HCINF1 (5, 30, 36). In patients with the p.Arg396Trp mutation, the arginine-to-tryptophane substitution leads to complete loss of catabolic activity of 24-hydroxylase due to destruction of the hydrogen bonds between arginine and the haem propionate group, thus blocking transient but crucial 24-hydroxylase substrate binding to haem (46, 47). Interestingly, the homozygous p.Arg396Trp mutation was also found in the cases described by David et al. and Collins et al. (38, 39). In most of the reported cases, the genetic panel was extended by the analysis of the genes associated with hyperparathyroidism, i.e., MEN1 (encoding the tumour suppressor protein menin; MEN1, Multiple Endocrine Neoplasia type 1), CaSR (encoding the G protein-coupled extracellular calcium-sensing receptor), HRPT2 (encoding the tumour suppressor parafibromin) and CDK (encoding the tumour suppressor cyclin-dependent kinase) (37, 48, 49). In the case reported by David et al., CaSR and MEN1 mutations, which are underlying causes of hyperparathyroidism, were excluded (38). In the case described by Collins et al., genetic testing confirmed that the patient was homozygous for the pathogenic variant c.1186C>T, p.Arg396Trp in the *CYP24A1* gene (39). (39) Documented cases of primary hyperparathyroidism and missense p.Arg396Trp mutation raise the question of whether the described genetic alteration predisposes patients to parathyroid autonomy. The link between loss-of-function *CYP24A1* mutations and the development of primary hyperparathyroidism has not been confirmed (50). In the present case, a heterozygous missense variant (rs114368325; p.Arg396Trp) was also identified, together with the second pathogenic variant. In this study, we were unable to confirm the heterozygosity or compound heterozygosity of both variants; therefore, we cannot discuss the recessive or dominant inheritance of the disease.

The coexistence of parathyroid autonomy and pathogenic variants in the *CYP24A1* gene is not associated only with p.Arg396Trp mutations. Loyer et al. reported two cases of concomitant PTH-dependent hypercalcaemia and deleterious compound heterozygous mutations in the gene encoding vitamin D 24-hydroxylase (37). In the first case, a 39-year-old female with persistent hypercalcaemia after the removal of hyperplastic parathyroid fluid, p.Cys380Arg/p. Leu409Ser compound heterozygous mutations were identified (37). Interestingly, in the same patient, an intronic HRPT2 gene polymorphism (c.1418-17C>G) was found (37). In the second case, genetic testing confirmed compound heterozygous (p.Leu335Profs11/.p.Arg396Gln) mutations in the *CYP24A1* gene, intronic polymorphisms (c.237 + 28 T>C) in the HRPT2 gene and the p.Gln1011Glu variant in the CaSR gene (37). Hypercalcaemia due to a novel heterozygous pathogenic variant (p.Pro392Argfs*9) in the CYP24A1 gene and concomitant primary hyperparathyroidism were documented in the case of a 23-year-old female by Liu et al. (40).

In PHPT, the only curative treatment is parathyroidectomy. In patients with proven *CYP*24A1 mutations, management should concentrate on eliminating or reducing hypercalcaemia and hypercalciuria. Thus, the basis of long-term strategies is a low vitamin D and calcium diet, in which vitamin D supplementation is omitted and sunlight exposure is avoided. In patients for whom this approach is not sufficient, various pharmacologic therapies have been described. Glucocorticoids that reduce enteral calcium absorption and inhibit the conversion of serum 25(OH)D_3_ to active 1,25(OH)_2_D_3_ were found to be ineffective in patients with *CYP24A1* defects (7, 51). However, effective therapeutic outcomes have been described in patients treated with imidazoles, such as ketoconazole or less toxic fluconazole, which reduce 1,25(OH)_2_D_3_ synthesis by inhibiting CYP enzymes (cytochrome enzymes) (25, 52).

The limitation of this study was the unavailability of patient family members, which allowed us to clearly determine whether the variants were located cis or trans within the *CYP24A1* gene and therefore how their concurrence affected the clinical outcome of the patient.

## Conclusions

The present case underlines the importance of accurate clinical evaluation of hypercalcaemia. Rarely may multiple causes of hypercalcaemia coexist, which complicates the diagnostic process. Identification of such conditions often requires a wider range of diagnostic techniques. In patients with PTH-independent hypercalcaemia, the measurement of vitamin D metabolites using LC–MS/MS analytical technique, followed by genetic testing (e.g., NGS technique), may help to identify carriers of *CYP24A1* mutations.

### Patient perspective

Currently, the patient reports an improvement in her wellbeing. The patient is pleased that the present treatment was limited by adequate hydration, the avoidance of sun exposure and the lack of vitamin D supplementation.

## Data availability statement

The datasets presented in this article are not readily available because of ethical and privacy restrictions. Requests to access the datasets should be directed to the corresponding authors.

## Ethics statement

Written informed consent was obtained from the individual(s) for the publication of any potentially identifiable images or data included in this article.

## Author contributions

DL: Project administration, Methodology, Writing – review & editing, Writing – original draft, Investigation, Data curation, Conceptualization. AS: Writing – review & editing, Writing – original draft, Investigation, Data curation, Conceptualization. JL: Writing – review & editing, Writing – original draft, Investigation, Data curation. MK: Writing – review & editing, Writing – original draft, Investigation, Data curation. MD: Writing – review & editing, Writing – original draft, Investigation, Data curation. AW: Writing – review & editing, Writing – original draft, Methodology, Investigation, Data curation. WM: Writing – review & editing, Writing – original draft, Supervision. WZ: Validation, Writing – review & editing, Writing – original draft, Supervision, Conceptualization. PG: Validation, Project administration, Writing – review & editing, Writing – original draft, Supervision, Methodology, Investigation, Conceptualization.
